# Supplementary material for: Reproducibility2020: Progress and priorities
Source: F1000Res. 2017 May 2;6:604. [Version 1] doi: 10.12688/f1000research.11334.1 (PMC5461896; doi:10.12688/f1000research.11334.1)
Supplement: Peer review history

# Peer review: Referee response for version 1

**DOI:** 10.5256/f1000research.12234.r22382

**Published:** 2017-06-01

**Authors:** Lenny Teytelman

**Affiliations:** 1 protocols.io, Berkeley, CA, USA

This is a carefully considered, well written, and comprehensive overview of the numerous causes of irreproducibility and the many ongoing efforts to address them. This manuscript also provides a set of useful actionable recommendations for researchers, funders, journals, and other stakeholders to improve the rigor and reproducibility of research.

 Below are specific comments that I hope the authors will find useful for revising and improving their paper.

  

1. ATCC is one of the main funders of GBSI and this report mentions ATCC a couple of times. The mentions are appropriate, but the GBSI/ATCC relationship should be clearly disclosed in the COI.
2. [Abstract and Introduction]
  Both the abstract and introduction mention the 2012 Amgen report as the beginning of attention to reproducibility. Without a doubt, the Amgen and Bayer headlines have led to a spike of attention and discussion; however the reproducibility issue is not a new problem. Inability to repeat the work of others is as old as science itself and much has been previously written regarding this issue (examples: https://www.ncbi.nlm.nih.gov/pubmed/16510544, http://www.the-scientist.com/?articles.view/articleNo/16604/title/Microarray-Data-Stands-Up-to-Scrutiny/, http://www.nature.com/ng/journal/v41/n2/full/ng.295.html, http://iai.asm.org/content/78/12/4972.full, http://journals.plos.org/plosmedicine/article?id=10.1371/journal.pmed.0040028, http://journals.plos.org/plosmedicine/article?id=10.1371/journal.pmed.0020272, http://elpub.scix.net/data/works/att/001_elpub2008.content.pdf). Moreover, many efforts to improve reproducibility are significantly older than 2012 (for example, Current Protocols, Open Wet Ware, Nature Protocol Exchange, JOVE, and more). Would be good to explicitly acknowledge this.
3. [Introduction] “ *Based on these findings, GBSI completed an economic study in 2015 and estimated that the prevalence of irreproducible preclinical research exceeds 50%, with associated annual costs of approximately $28B in the United States alone[4].”*
  As has been publicly discussed after the PLOS Biology publication [4], the estimate of $28B cost of irreproducible research is on shaky ground (see The Sensational vs. the Useful in the Quest for Reproducibility in Research and Study claims $28 billion a year spent on irreproducible biomedical research). It extrapolates to all of US Biomedical Funding from a few estimates of irreproducibility in specific fields. I know of no quantitative research that evaluates reproducibility of published basic research in zebra fish or drosophila communities. If reproducibility problems are greater in cancer, human cell lines, and other research fields, the overall scale of the reproducibility problem across all biomedical research could be smaller.
  Also, I very much appreciate the authors’ note that the “irreproducible” definition is tricky and that they include results, methods, and inferential reproducibility in their analysis. So, the results may be simply “hard to reproduce” due to missing details or reagents, but they would be included in the “irreproducible total”. The definition issues further complicate the attempt to estimate in dollar amounts the scale of irreproducible research.
  Instead of saying, “the prevalence of irreproducible preclinical research exceeds 50%, with associated annual costs of approximately $28B in the United States alone”, I urge the authors to simply refer to their publication with something more general such as, “GBSI’s 2015 economic study highlighted the high level of economic costs from poor reproducibility.”
4. [Study design and analysis]
  Box 2 recommends online training courses as highly cost-effective. It is true that they are cost effective, but are they effective when it comes to improving study design? Given how busy scientists tend to be, it is unclear that they will actually devote time to watching online training videos. (For example, podcasts for scientists tend to be consumed much more readily than videos of the same length, as people can listen during commute, runs, cooking, etc. In contrast, videos longer than 3-4 minutes are barely watched by anyone to the end.)
5. [Laboratory protocols]
  This section should probably mention the Protocol Exchange from *Nature/Springer *which is a protocol repository that was started over a decade ago to improve the reporting of methods.
  The authors might also want to include a mention of Bio-protocol, a journal devoted to increasing reproducibility. Though a selective peer-reviewed journal rather than a repository, Bio-protocol is also connecting to journals and eLife recently included them in their author guidelines to encourage scientists when appropriate to submit new method details to Bio-protocol in parallel with their eLife manuscript submission.
6. [Reporting and review]
  In the data reporting section, I recommend adding a brief discussion of data repositories such at Dryad and figshare. Journal policies regarding data sharing are critical and this overview of the genomics community journal policies from Heather Piwowar and Wendy Chapman is relevant: http://elpub.scix.net/data/works/att/001_elpub2008.content.pdf.
  Also, the explicit data policy from the Public Library of Science is an important step in improving reproducibility of published work.
  Related to the data policies, sharing code and software from computational pipelines used to analyze the data is critical. Perhaps add a mention of policies encouraging proper reporting and sharing of code/software?
7. [Reporting and review]
  There are important experiments happening with open review from publishers such as F1000 Research, EMBO, BMJ, PeerJ and others. Transparent publication with review/author response history can be helpful for reproducibility as readers can see reviewers’ concerns and that can help to discern which parts of the paper are more or less trustworthy.
  Another relevant proposal is for the adoption of CRediT (Contributor Roles Taxonomy) by publishers. (See Transparency In Authors' Contributions And Responsibilities To Promote Integrity In Scientific Publication.)
8. [Reporting and review: open access policies]
  This section does a good job of summarizing open access initiatives and policies from funders, but the link to reproducibility is unclear.  As an advocate for open access, I am delighted to see these developments, but the connection between open access publishing and increased reproducibility is not obvious to me.
  A paper in a subscription journal can be solid and reproducible, while one in an open access journal is not. The reverse is just as likely. Certainly, this is more a function of chance and editorial and peer review vigilance than the journal’s business model.
  An argument can be made for how open access enables reproducibility initiatives (ex. CiteAb), but I don’t think I saw it in this paper.
9. [Reporting and review: preprints]
  As above for open access, I am a huge fan of preprints but am unsure how they fit into the push for greater reproducibility.  Preprints, of course, shorten publication delays, facilitating communication and speeding up research. However, preprints are not peer-reviewed, do not go through conflict-of-interest checks, data/method reporting compliance checks, and so forth. At scale adoption of preprints in biology is welcome for many reasons, but not exactly due to more rigor and higher reproducibility.
  (Possibly, preprints reduce the pressure to publish and create a track record of a paper’s initial state, reducing publication biases? Preprints can also help to challenge previously-published work and to report negative results. If these are the arguments for preprints improving reproducibility, please make this case explicitly in the manuscript.)
  (Minor note: the use of “preprint” versus “pre-print” is inconsistent in this paper. Please remove the extra dash.)
10. [Table 3, action plan]
*For funders*, there is a recommendation to “ *Enact policies requiring study design pre-registration*”. I am on the steering committee for COS’s pre-registration initiative and support this effort, but I am not sure that “requiring” pre-registration widely is appropriate. This will depend on the funder and specific research grant. For example, in the case of method development and highly explorative grants, pre-registration is unlikely to be productive. How about “encourage where appropriate” instead of “require”?
  For journals, there is a recommendation to “ *Require authors to link to version-controlled protocols*”. Again, “require” is a strong term. In certain cases, it may be better to share a protocol directly as part of the publication (for example, JOVE). A more general “encourage or require detailed reporting of protocols” may be more appropriate.
11. [Conclusion] *“Irreproducibility is a serious and costly problem in the life sciences. Measured reproducibility rates are shockingly low, requiring significant effort to solve this problem.” *
  I very much agree with the first sentence in that irreproducibility is a serious problem. However, is the reproducibility rate “shockingly” low? What is that rate for biology in general? As discussed above, 50% may be the number for some fields but not for others. More importantly, what rate are we aiming for? 70%? 90%? If all of the action items recommended in this report were followed, what rate would we end up with? Is our current level of reproducibility better or worse than it was 30 years ago? What is the optimal reproducibility rate from society’s perspective?
  I don’t have the answers to the above questions. We need a lot more data to make informed statements about the levels of reproducibility over time. It is terrific that we are discussing this issue and the initiatives to address the problem, but I urge caution in editorializing about whether today’s reproducibility levels are a “crisis” or are “shocking”. Science is hard and because it is pushing the boundaries of knowledge, we will never be at 100% of published research being reproducible. We can and should do a lot better, hence all of the initiatives, but it will never be 100%.
12. [General thoughts]
  As I mention in #11 above, with the exception of a few efforts from Science Exchange and the Center for Open Science, we have very little data on the reproducibility issue. The authors may want to include in their discussion the need for more quantitative studies about replication and reproducibility over time. We need ways to assess the various initiatives and to measure whether they are in fact improving the overall reproducibility levels of published research.
  Also, most of the recommendations and discussion in this Report are focused on the design, execution, and publication steps of the research cycle. However, given the complexity of research and the fact that we will never attain 100% reproducibility, efforts aimed at post-publication opportunities to improve reproducibility may be particularly effective. Perhaps we should pay more attention not just to preventing mistakes, but to ways to correct and improve papers, long after publication.
  This Report mentions post-publication review and retractions, but there are other promising efforts in this phase. Versioning, as implemented on F1000Research and bioRxiv, has great potential. There is a need for technologies that automatically connect readers to corrections and discussion on the papers that they have in their libraries. Crossmark from Crossref is a great initiative aimed at making corrections discoverable. Also, an interesting recent proposal argues for rethinking of “retractions/corrections” in favor of "amendments" to increase post-publication evolution and improvement of work.

-------------------------------------

 I would like to stress that I thoroughly enjoyed this report and am grateful to the authors and GBSI for their efforts to improve the research enterprise for the benefit of scientists and the public. The authors should feel free to ignore any of the above suggestions if they disagree.

I have read this submission. I believe that I have an appropriate level of expertise to confirm that it is of an acceptable scientific standard.

# Peer review: Referee response for version 1

**DOI:** 10.5256/f1000research.12234.r22387

**Published:** 2017-05-30

**Authors:** Michael S. Lauer

**Affiliations:** 1 National Institutes of Health (NIH), Bethesda, MD, USA

Freedman and colleagues present a narrative review on current efforts underway to improve reproducibility in preclinical biomedical research.  They begin by summarizing the extent of the problem and noting that quality checkpoints are either used in disparate points of the research cycle or used only sparingly.  They identify key sources of irreproducibility as poor study design and analysis, inadequately authenticated reagents and reference materials, inadequately documented laboratory protocols, and inadequate reporting and review.   They describe the important roles of many stakeholders, including funders, researchers, research institutions, industry, foundations, professional societies, and the public. 

 The authors proceed to describe many efforts already under way including new funder requirements, journal guidelines, enhanced training opportunities, programs to enhance standards development and authentication checks, protocol repositories, improved reporting platforms, open access policies (including open access publishing, greater use of preprint servers, data and code sharing), data standards, and post-publication review.  They conclude with a “path forward” that they call the “Reproducibility2020 Action Plan” that includes specific recommendations for funders, researchers, institutions, journals, industry, foundations, and the public.

 Thoughts and comments:

  

- The paper is interesting, well-written, and well-documented.  I appreciated the many web links that take the reader directly to interesting sites.
- The authors suggest that the current crisis begins with the Amgen findings (Reference 2).  While that was a defining moment, I wonder whether it’s also worth mentioning that contemporary discussion about false research findings dates back at least as far back as Ioannidis 2005 ( https://doi.org/10.1371/journal.pmed.0020124).  Ioannidis there suggests that exploratory research was highly vulnerable because of small sample sizes, overly flexible designs, and biased designs (e.g. with lack of randomization and proper masking).
- Table 1: I commend the authors for noting that “the chance of an irreproducible finding is much higher than the commonly noted 5% threshold.”  This is widely under-appreciated, even by well-trained scientists.  The authors might consider spelling out that prospective, properly done sample size calculations are critical to overcoming this problem.  The “elephant in the room” is that sample sizes will have to increase substantially, meaning that with constrained funds researchers will be forced to conduct fewer experiments.  But as some have noted ( Cressey D, Nature, April 15, 2015), that may be good for the enterprise – it would be better to do fewer properly powered experiments than to do too many woefully underpowered experiments.
- Table 1 and elsewhere: Should there be a “Consumer Reports” for antibodies, cell lines, and other resources?  Or maybe I’m missing it, and you’re saying that’s happening.  Such a “Consumer Reports” would allow for large-scale surveys in which researchers can report problems with purchased materials.
- Table 1: Another potential solution to study design and analysis is mandatory sharing of statistical code (e.g. in SAS, R, or Stata).  This is already common practice in some fields (e.g. economics).
- Table 2: Another consequence for the public is lack of faith in science.  They hear scientists promising the moon, and then nothing happens. 
- Table 2: There is an ethical problem subjecting animals and people to inadequately designed or documented experiments that were doomed to be irreproducible from the beginning.
- Table 2 or elsewhere: NAS just released a report on research integrity in which notes a continuum between frank misconduct (fabrication, falsification, and plagiarism) and “practices detrimental to research.”  The authors might want to consider the comments of the report ( https://www.nap.edu/catalog/21896/fostering-integrity-in-research). 
- There have been some recent successes in improved rigor, such as in preclinical stroke research.  (For example, see http://circres.ahajournals.org/content/early/2017/04/04/CIRCRESAHA.117.310628).  The authors note that “stroke research has uniquely improved.”
- Page 6 – the link didn’t take me directly to “Statcheck software,” though I did eventually find it.
- Protocols – many leading clinical journals require authors to submit full clinical trial protocols along with the manuscripts.
- Table 3
  - Should it be the responsibility of funders to provide statistical consultation to applicants?
  - Should it be the responsibility of funders to pay for open access and transparency tools? 
  - Should funders include dedicated reviews on methodological issues for those applications deemed meritorious by content? 

I have read this submission. I believe that I have an appropriate level of expertise to confirm that it is of an acceptable scientific standard.
